# Frequency-dependent changes in the amplitude of low-frequency fluctuations in internet gaming disorder

**DOI:** 10.3389/fpsyg.2015.01471

**Published:** 2015-09-28

**Authors:** Xiao Lin, Xize Jia, Yu-Feng Zang, Guangheng Dong

**Affiliations:** ^1^Department of Psychology, Zhejiang Normal University, JinhuaChina; ^2^Peking-Tsinghua Center for Life Sciences, Peking University, BeijingChina; ^3^Cognitive and Brain Disease Research Center, Hangzhou Normal University, HangzhouChina; ^4^Zhejiang Key Laboratory for Research in Assessment of Cognitive Impairments, Hangzhou Normal University, HangzhouChina; ^5^Institute of Psychological Research, Zhejiang Normal University, JinhuaChina

**Keywords:** internet gaming disorder, resting-state functional magnetic resonance imaging, amplitude of low-frequency fluctuation

## Abstract

Neuroimaging studies have revealed that the task-related functional brain activities are impaired in internet gaming disorder (IGD) subjects. However, little is known about the alternations in spontaneous brain activities about them. Recent studies have proposed that the brain activities of different frequency ranges are generated by different nervous activities and have different physiological and psychological functions. Thus, in this study, we set to explore the spontaneous brain activities in IGD subjects by measuring the fractional amplitude of low-frequency fluctuation (fALFF), to investigate band-specific changes of resting-state fALFF. We subdivided the frequency range into five bands based on literatures. Comparing to healthy controls, the IGD group showed decreased fALFF values in the cerebellum posterior lobe and increased fALFF values in superior temporal gyrus. Significant interactions between frequency bands and groups were found in the cerebellum, the anterior cingulate, the lingual gyrus, the middle temporal gyrus, and the middle frontal gyrus. Those brain regions are proved related to the executive function and decision-making. These results revealed the changed spontaneous brain activity of IGD, which contributed to understanding the underlying pathophysiology of IGD.

## Introduction

Internet addiction disorder (IAD) has been defined as the individual’s inability to control the excessive use of the Internet, even in the face of the negative consequences to psychological functioning aspects (Young, 1998; Fitzpatrick, 2008; Tao et al., 2008; Flisher, 2010). It has been proposed as a “behavioral addiction” according to its negative effects on social mental health (Kuss and Griffiths, 2012). However, little is known about the mechanism of IAD, and a uniformly definition of IAD hasn’t been formed and the Diagnostic and Statistical Manual 4 (DSM-4) didn’t include this behavioral disorder (Block, 2008). Along with the rapid spread of IAD, the DSM-5 is developed for internet gaming disorder (IGD) based on the definition of substance-use disorders and addictions (Frances and Widiger, 2012; American Psychiatric Association, 2013; Petry and O’Brien, 2013; Petry et al., 2014).

There are many different types of IAD due to the internet’s diverse functions. In general, IAD consists of three subtypes: IGD, Internet pornography, and e-mailing (Block, 2007). Considering the definition of addiction, all these categories of IAD share four defining characteristics: excessive use, withdrawal, tolerance, and negative repercussions (Beard and Wolf, 2001; Block, 2008; Tao et al., 2010). As the most prevalent form of IAD (Dong et al., 2012a), IGD may share specific neuropsychological characteristics with other behavioral addictions, such as pathological gambling (Griffiths, 2005; Grant et al., 2010; Dong et al., 2012b; Han et al., 2012; Dong and Potenza, 2014).

Numerous imaging studies have investigated the characteristics of IGD using different tasks (Fowler et al., 2007; Dong et al., 2011a, 2012b; Han et al., 2011a; Xu, 2013), but it is difficult to compare data obtained from different experimental paradigms and draw clinically helpful conclusions from different cognitive tasks (Zang et al., 2007a). Resting-state fMRI studies have revealed some abnormalities of the brain activation in IGD (find more descriptions from a review by Weinstein and Lejoyeux (2015). IGD subjects have higher impulsiveness, which is a typical symptom of drug addiction; this symptom is related to the decreased activation of cingulate gyrus, which involves cognitive control (Dong et al., 2012a). An fMRI study also showed enhanced regional homogeneity (ReHo) in the brainstem, inferior parietal lobule, left posterior cerebellum, and left middle frontal gyrus that are related with sensory-motor coordination which might be relevant to the finger movement of playing internet games (Dong et al., 2012c).

Resting-state fMRI has been developed as a new technique since the Biswal’s study (Biswal et al., 1995). They first reported the highly synchronous spontaneous low frequency (0.01–0.08 Hz) fluctuations in BOLD signal among motor cortices, concluding the amplitude of low-frequency fluctuation (ALFF) was a neurophysiologic indicator (Biswal et al., 1995). On the basis of ALFF, Zang et al. (2007b) promoted another tool to depict local brain activity - the fractional amplitude of low-frequency fluctuation (fALFF), which could detect the regional intensity of spontaneous fluctuations in BOLD signal (Zou et al., 2008; Zuo et al., 2010). Recently, fALFF was broadly used in mental disorder patients’ studies, such as depression (Guo et al., 2013), schizophrenia (Bluhm et al., 2007), attention deficit hyperactivity disorder (Zang et al., 2007b), IGD (Yuan et al., 2013), and so on. It is still unclear whether the brain activity abnormalities of IGD are related to specific frequency bands. It is important to detect brain spontaneous fluctuations at specific frequency more than a broad frequency band. There are many diverse oscillations in the brain, the frequencies of them are ranging from very slow oscillations with periods of tens of seconds to very fast oscillations with frequencies exceeding 1000 Hz (Bullock, 1997). Buzsáki and Draguhn (2004) proposed an ‘oscillation class’ which contains 10 frequency bands extending from 0.02 to 600 Hz (Penttonen and Buzsáki, 2003). And Zuo et al. (2010) investigated the fALFF at four frequency bands and found that the oscillations are linked with specific neural processes (Buzsáki and Draguhn, 2004; Knyazev, 2007). They found that amplitudes of oscillations (0.01–0.027 Hz) at low frequency were most robust in the cortical structures and high frequencies were most robust in the subcortical structures such as the basal ganglia. Studies have revealed that schizophrenia patients had particular abnormalities of oscillations amplitudes in the slow-4 frequency band (Yu et al., 2014). Han et al. (2011b) also proved that abnormalities of brain function in amnestic mild cognitive impairment patients exposed different activation patterns in different frequency bands.

In the present study, we collected fALFF values of the frequency across 0–0.25, including six frequency bands of 0–0.01 Hz, 0.01–0.027 Hz, 0.027–0.073 Hz, 0.073–0.198 Hz, and 0.198–0.25 Hz in IGD, according to Buzsáki’s “oscillation classes”. We sought to compare the fALFF value between IGD and HC in different bands and address two issues: first, whether the IGD subjects show abnormal fALFF amplitudes when compare to healthy controls; second, whether the abnormalities of IGD are associated with specific frequency bands.

## Materials and Methods

### Participant Selection

The experiment conforms to The Code of Ethics of the World Medical Association (Declaration of Helsinki) and is approved by the Human Investigations Committee of Zhejiang Normal University. Fifty-two university students were recruited through advertisements [26 IGD, 26 healthy controls (HC)]. They were all right-handed males. IGD and HC groups did not significantly differ in age (IGD: *N* = 26, 22.2 ± 3.13 years; HC: *N* = 26, 22.28 ± 2.54 years; *t*(50) = 0.1, *p* = 0.9). Because of the higher IGD proportions among men, only males were included. Participants were required to sign the informed consent and all participants went through structured psychiatric interviews (M.I.N.I.) (Lecrubier et al., 1997) performed by an experienced psychiatrist with an administration time of approximately 15 min. All participants were free of Axis I psychiatric disorders listed in MINI. All the participants did not meet DSM-4 criteria for drug abuse or dependences, including alcohol, although all IGD and HC participants reported alcohol consuming in their lifetime. All participants were instructed not to use any substances, including coffee, tea, on the day of scanning. No participants reported brain damages or previous experience with illicit drugs (e.g., cocaine, marijuana).

The diagnosis of IGD was determined based on scores of 50 or higher on Young’s online Internet Addiction Test (Young, 1998). As a special behavior addiction, the operational definition and diagnostic standards for IGD are still inconsistent. In the present study, the IGD group was composed of individuals who met the general IAD criteria (scores over 50 in the IAT) and reported “spending most of their online time playing online games (>80%)” (Blaszczynski, 2008; Weng et al., 2013). The IAT score of IGD group (72 ± 11.7) was much higher than the healthy controls [29 ± 10.4), *t*(50) = 14, *p* = 0.000].

### Data Acquisition

After conventional localizer scanning, the T1-weighted images were obtained with a spoiled gradient recall sequence [TR = 240 ms; echo time (TE) = 2.46 ms; flip angle (FA) = 90°; field of view (FOV) = 220~220 mm^2^; data matrix = 256~256]. Then, resting-state functional images were acquired using an echo-planar-imaging sequence (TR = 2000 ms; TE = 30 ms; FA = 90°; FOV = 220~220 mm^2^; data matrix = 64~64) with 33 axial slices (slice thickness = 3 mm and slice gap = 1 mm, total volumes = 210) in one run of 7 min. The subjects were required to keep still and not think about anything systematically during the scanning. At the end of the data acquisition, all subjects confirmed that they remained awake during the whole scanning period.

### Data Preprocessing and fALFF Calculation

All of the functional image processing was performed with Data Processing Assistant for Resting-State fMRI [DPARSF (Yan and Zang, 2010)^1^] software. For each participant, the first 10 time points were excluded from further analysis, which is to avoid transient signal changes before magnetization reached steady state and to allow subjects to get used to the fMRI-scanning environment. The remaining 200 brain volumes were corrected for slice timing and realigned for head movement correction. Only participants with head motion less than 1.5 mm in the x, y, or z direction and less than 2 rotation about each axis were included. 26 HC and 26 IGD subjects were valid in the present study. Then, all of the realigned images were spatially normalized, and then resampled to 3 mm isotropic voxels and spatially smoothed (full-width at half-maximum = 6 mm), and the linear trend was removed. After preprocessing, fALFF was calculated using DPARSF. Briefly, for a given voxel, the time series was first converted to the frequency domain using a “fast Fourier transform.” The square root of the power spectrum was computed and then averaged across a predefined frequency interval. This averaged square root was termed fALFF at the given voxel of predefined frequency bands (Zang et al., 2007a). We divided the full frequency range (0–0.25 Hz) into five sub-bands: slow-6 (0–0.01 Hz), slow-5 (0.01–0.027 Hz), slow-4 (0.027–0.073 Hz), slow-3 (0.073–0.198 Hz), and slow-2 (0.198–0.25 Hz) (35, 46, 30), and computed fALFF of each frequency bands.

### Statistical Analysis

A two-way (group and frequency band) repeated-measures analysis of variance (ANOVA) was performed on a voxel-by-voxel basis with group (IGD and HC) as a between-subject factor and frequency band (slow-2, slow-3, slow-4, slow-5, slow-6) as repeated-measures. We also calculated the ROI-based correlation analysis following up the significant main effect and interaction between the severity of IGD and the fALFF values, and we picked fALFF values from specific bands.

## Results

**FIGURE 1:**
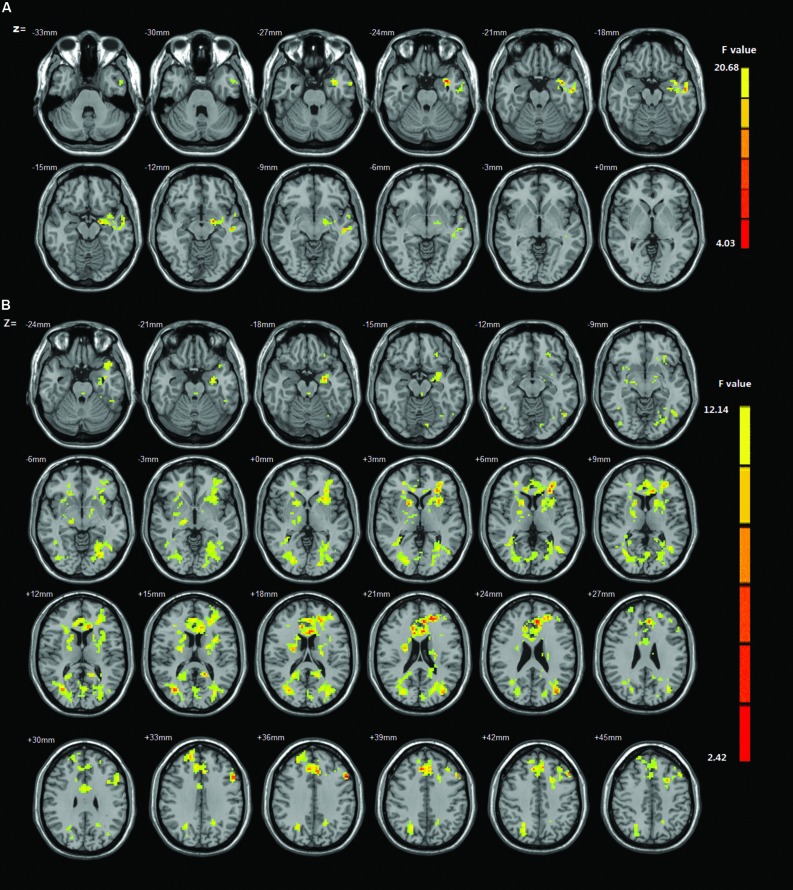
**(A)** The main effect for group on amplitude of low-frequency fluctuation (ALFF). Brain regions in which the fractional amplitude of low-frequency fluctuation (fALFF) is different between Internet gaming disorder (IGD) and healthy controls. The IGD subjects showed increased ALFF in warm colored brain regions especially the cerebellum, whereas decreased ALFF in cool colored regions including the superior temporal gyrus. Map threshold of multiple comparisons were set at *p* < 0.05 using AlphaSim correction. **(B)** The interaction between frequency band and group on fALFF. The results were obtained by a two-way repeated-measures analysis of variance (ANOVA) calculated by AFNI.

**FIGURE 2:**
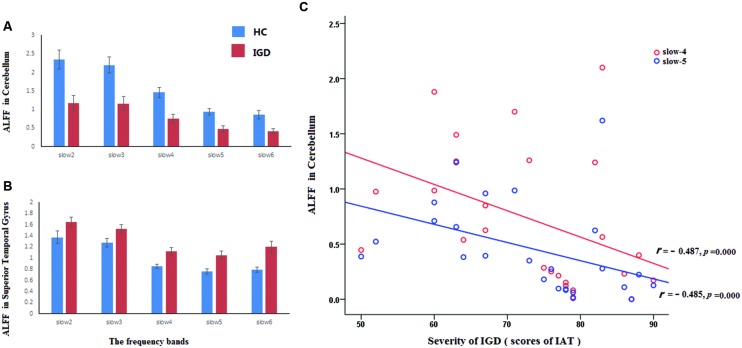
**The ALFF values in superior temporal gyrus and the cerebellum.** The red and blue rectangle represented IGD subjects and healthy controls, respectively. The full frequency band (0–0.25 Hz) was divided into five bands. They were displayed in **(A,B)** [slow-6 (0–0.01 Hz), slow-5 (0.01–0.027 Hz), slow-4 (0.027–0.073 Hz), slow-3 (0.073–0.198 Hz), and slow-2 (0.198–0.25 Hz)]. **(C)** Reveled the ROI-based correlation between the severity of IGD and the ALFF values in the cerebellum, the red and blue line represented frequency slow-4 and slow-5, respectively.

Main effects from the two-way repeated-measures ANOVA were shown in **Figure 1**, **Tables 1** and **2**. We used Alphasim correction for the multiple comparisons in imaging data. The corrected *p* < 0.05 corresponds to a combination of uncorrected *p* < 0.05 and cluster size >248 mm^3^). ROI based correlation analysis was carried out between fALFF values and the severity of IGD (scores of IAT). The cerebellum showed significant negative correlation with IGD severity (slow-4: *r* = -0.487, *p* = 0.000; slow-5: *r* = -0.485, *p* = 0.000; see **Figure 2C**). The coordinate of ROI was defined by the activation peak of the survived cluster. The radius of ROI is 4 mm, and is made by the software REST^2^.

**Table 1:** Brain regions with a main effect of group.

Region	BA	size	*x*	*y*	*z*	
Superior temporal gyrus	41,42	235	-33	3	-24	IGD<HC
Cerebellum		1180	0	-12	-51	IGD>HC

*We list significant clusters of main effect of group. Shown are the Brodmann Area, the size of the cluster, the coordinates of the local maxima (in MNI space), and which group have the higher fractional amplitude of low-frequency fluctuation (fALFF) values. If multiple local maxima existed in the same region, only the maximum with the highest *F* score is shown.*

**Table 2:** Brain regions with interaction effect between group and frequency.

Region	BA	size	*x*	*y*	*z*
Left cerebellum		1348	-21	-51	-45
Bilateral anterior cingulate	24,32	1947	-6	36	24
Left lingual gyrus	18	680	-36	-78	24
Right middle temporal gyrus	21	648	-35	-75	15
Left middle frontal gyrus	46	264	-54	18	36

*We list significant clusters of interaction effect between group and frequency. Shown are the Brodmann Area, the size of the cluster, the coordinates of the local maxima (in MNI space). If multiple local maxima existed in the same region, only the maximum with the highest *F* score is shown.*

**FIGURE 3:**
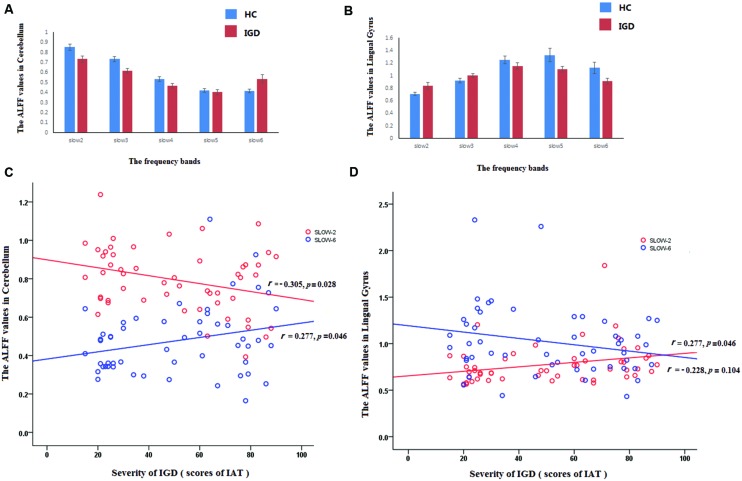
**Reverse pattern in cerebellum and the lingual gyrus at different bands in IGD.** The red and blue rectangle represented IGD subjects and healthy controls, respectively. The full frequency band (0–0.25 Hz) was divided into five bands. They were displayed in **(A,B)** [slow-6 (0–0.01 Hz), slow-5 (0.01–0.027 Hz), slow-4 (0.027–0.073 Hz), slow-3 (0.073–0.198 Hz), and slow-2 (0.198–0.25 Hz)]. **(C)** revealed the reversed correlation between the fALFF of the cerebellum and the severity of IGD for different frequency (slow-2: *r* = -0.305, *p* = 0.028; slow-6: *r* = 0.277, *p* = 0.046), **(D)** revealed the reversed correlation between the fALFF of the lingual cyrus and the severity of IGD for different frequency (slow-2: *r* = 0.277, *p* = 0.016; slow-6: *r* = -0.228, *p* = 0.104).

Significant interactions between frequency band and group were observed in the cerebellum, the anterior cingulate, the lingual gyrus, the middle temporal gyrus, and the middle frontal gyrus. The middle frontal gyrus showed increased amplitude values and the middle temporal gyrus showed decreased amplitude values in IGD. In addition, ROI-based analyses presented dynamic alteration of fALFF in the cerebellum and lingual gyrus along with frequency adaption (see **Figure 3**). In IGD, the cerebellum showed decreased amplitude values in the higher frequency realm (slow-2, slow-3, slow-4) and increased amplitude values in the lower frequency realm (slow-6, see **Figure 3A**). Conversely, lingual gyrus showed increased amplitude values in the higher frequency realm (slow-2, slow-3) and decreased amplitude values in the lower frequency realm (slow-6, see **Figure 3B**). These two regions shared a transition point at slow-5 band for the alteration of amplitude.

## Discussion

The present study investigated the abnormal spontaneous brain activity in IGD with the fALFF at different frequency bands. Main group effect revealed that the IGD demonstrated lower fALFF values in superior temporal gyrus and higher fALFF values in cerebellum. We presented BOLD fluctuation amplitudes in the whole frequency bands (0–0.25 Hz) and found a reversed pattern of changes in frequency realm in the cerebellum and lingual gyrus in IGD. These findings provide a full view of fALFF analyzes in frequency domain, and emphasize the importance of the selection of specific frequency for detecting abnormality related mental disorders.

### Different fALFF in Cortical between IGD and HC (The Main Effect of Group)

Previous literatures believed that the signal of slow-2 reflects very low frequency drift, and the slow-6 reflects high-frequency physiological noises (Zang et al., 2007a; Xu, 2013). The analysis of main effect of group focused on the spontaneous neural activity at specific frequency bands (slow-4 and slow-5) in IGD. The main effect of group revealed that the IGD showed lower fALFF values at slow-4 and slow-5 in cerebellum. A negative correlation between the fALFF values in cerebellum and the severity of IGD was found in the present study. The cerebellum is commonly classified as a motor structure whose function is not confined to movement coordination or balance and it also plays an important role in higher cognitive processes (De Zeeuw et al., 2011; Stoodley et al., 2012). Evidence from anatomical, physiological, and functional imaging studies has proved that people with lesions to the cerebellum showed deficiency of cognitive executive functions and working memory (Raymond et al., 1996; De Smet et al., 2013). It receives input from sensory systems and other brain areas, and integrates these inputs to adjust motor activity (Doyon et al., 2003; Ito, 2006; Yuan et al., 2011). The potential role of the cerebellum in addiction has been addressed in a recent paper, which proposed that the cerebellum is a potential regulation center that is impacted by addiction (Moulton et al., 2013). Literatures have demonstrated that IGD subjects are associated with greater-than-normal ReHo (Liu et al., 2010; Dong et al., 2012c) and functional connectivity (Ding et al., 2013) over the cerebellum. In the present study, a negative correlation between the fALFF values in cerebellum and the severity of IGD was observed (see **Figure 2C**), which also supports that the abnormal spontaneous neuronal activity in cerebellum is related with the inappropriate behavior of IGD.

The fALFF values were higher in superior temporal gyrus in IGD. Previous study showed that the IGD, compared to HC, showed decreased functional connectivity in the temporal area (Ding et al., 2013). Our previous study found decreased ReHo in the inferior temporal gyrus, and we infer it might be the results of a long duration of game playing (Dong et al., 2012c). The current findings are partially inconsistent with previous study, so we bring forward the hypothesis that increased fALFF in superior temporal gyrus may reflect higher level of brain activity correlating with the flexibility of movement in IGD, but the function of this area need further study.

### Frequency Dependent Amplitude Changes in IGD

The interaction effects between groups and frequency bands were observed in the cerebellum, the anterior cingulate gyrus, the lingual gyrus, the middle temporal gyrus, and the middle frontal gyrus.

#### Higher fALFF Values in Middle Frontal Gyrus in IGD

In the present study, the IGD participants showed higher fALFF values in left middle frontal gyrus at different bands. The middle frontal gyrus plays an important role in coordinate different systems, such as learning and memory, which is strongly related to mental operations (Cardinal, 2006). In a previous study, we concluded that IGD subjects show enhanced synchronization in sensory-motor coordination related brain regions (Van Rooij et al., 2011) – the online game playing requires players to integrate several systems, including the sensory system, motor control, motor coordinate, and information processing system (Ito, 2006). The current findings also support this assumption. This result is also consistent with Liu’s study (Liu et al., 2010), which found that subjects with IGD showed a significant increase in ReHo values in left middle frontal gyrus. So we draw the conclusion that the IGD participants showed higher fALFF values in left middle frontal gyrus, which might associate with the enhanced sensory-motor coordinate ability.

#### The Abnormality in Anterior Cingulate Gyrus in IGD

We found lower fALFF in anterior cingulate Gyrus at slow-6. The anterior cingulate region has been implicated in inhibition, controlling, and conflict monitoring (Paus, 2001; Goldstein et al., 2007) and the abnormalities have been mentioned in previous IGD studies (Liu et al., 2010; Moulton et al., 2013). As mentioned in introduction, the lower fALFF values may relate to decreased coordinating capability of long-distance neural activity. This assumption is supported by studies in this field: with a functional connectivity approach. Hong et al. (2013) reported reduced functional connectivity between ACC and PFC in IAD. Jiang et al. (2011) have proposed that the lower activities in the ACC may reflect the abnormal decreased spontaneous neuronal activity in this region and a functional deficit. Other task related studies have proved this point that the IGD always accompanied with cognitive dysfunctions, such as cognitive function deficiency (Dong et al., 2010, 2011b). So we believe the abnormality in ACC is related to the cognitive dysfunctions of IGD.

#### Reverse Pattern in Cerebellum and the Lingual Gyrus at Different Bands in IGD

It is important to note that the abnormalities of spontaneous neural activity in the IGD are dependent on specific frequency bands, especially in the cerebellum and the lingual gyrus. Comparing with the HC, the IGD showed decreased amplitude in the lower frequency bands (slow-4, slow-5, slow-6) and increased amplitude in the higher frequency bands (slow-2, slow-3) in the lingual gyrus. On the contrary, the IGD showed increased amplitude in the lower frequency bands (slow-6) and decreased amplitude in the higher bands (slow-2, slow-3, slow-4) in the cerebellum (**Figures 2A,B**). It has been revealed that different oscillatory bands are developed by different mechanisms and have different physiological functions (Bullock, 1997; Yuan et al., 2013). As previous studies have proved that the lower frequency fluctuations possess higher magnitude power and the higher frequency fluctuations have lower magnitude power (Baria et al., 2011; Yuan et al., 2013). The current findings might suggest that the IGD have increased coordinating capability of long-distance neural activity in the cerebellum and in the lingual gyrus. This assumption can be supported by previous study which reported that subjects with IGD exhibited increased functional connectivity in the bilateral cerebellum (Liu et al., 2010; Ko, 2014), and another study have detected gray matter density deficits in lingual gyrus which may relate to long-distance neural activity (Weng et al., 2013).

## Conclusion

The findings in the present study suggested that the IGD subjects showed abnormal fALFF in many brain regions, including the cerebellum (IGD < HC) and the superior temporal gyrus (IGD > HC). The present study can help to understand the pathophysiology of IGD and the full frequency amplitude analysis may potentially help to select specific frequency range for detecting IGD-related brain activities.

## Author Contributions

XL analyzed the data, wrote the first draft of the manuscript; XJ contributed to data analyze, Y-FZ contributed to the guidance of Experimental methods, and improved the manuscript. GD designed this research, revised and improved the manuscript. All authors contributed to and have approved the final manuscript.

## Conflict of Interest Statement

The authors declare that the research was conducted in the absence of any commercial or financial relationships that could be construed as a potential conflict of interest.

## Footnotes

1 http://www.restfmri.net

2 www.restfmri.net
